# Bilateral implantation of extended depth of focus intraocular lens in patients with early or intermediate age-related macular degeneration

**DOI:** 10.1007/s10792-026-03973-4

**Published:** 2026-01-30

**Authors:** Nicolò Ciarmatori, Antonio Cartabellotta, Ginevra Giovanna Adamo, Camilla Biffi, Pietro Maria Talli, Marco Pellegrini, Marco Mura

**Affiliations:** 1 Department of Translational Medicine, University of Ferrara, Ferrara, Italy; 2 Sant’Anna University Hospital, Via Aldo Moro 8, 44124 Cona (Ferrara), Italy; 3 King Khaled Eye Specialist Hospital, Riyadh, Saudi Arabia

**Keywords:** Cataract, Age-related macular degeneration, Extended depth of focus intraocular Lens, Retina

## Abstract

**Purpose:**

To evaluate the visual outcomes, dysphotopsia profile, and patient satisfaction following bilateral implantation of the AcrySof IQ Vivity intraocular lens (IOL) in patients with early or intermediate age-related macular degeneration (AMD).

**Methods:**

Prospective, single-center study. 24 patients (48 eyes) with bilateral cataract and early or intermediate AMD who underwent bilateral implantation of the AcrySof IQ Vivity IOL. The primary outcome was monocular corrected distance visual acuity (mCDVA). Secondary endpoints were distance-corrected and unaided monocular and binocular visual acuity at 4 m, 66 cm, and 40 cm, monocular defocus curve, subjective dysphotopsia (McAlinden QoV questionnaire) and haloes perception (Aston Halometer), visual function (Catquest-9SF), and spectacle independence (IOLSAT).

**Results:**

At 3 months postoperatively, mCDVA improved significantly from 0.30 to 0.00 logMAR (p < 0.001). Binocular uncorrected distance and intermediate visual acuities of ≤ 0.2 logMAR were achieved by 23 (95.8%) and 17 (70.8%) patients, respectively. The defocus curve showed visual acuity of 0.2 logMAR across a range of + 1.40 D to -1.72 D. Bothersome dysphotopsias significantly decreased, with QoV scores improving from 58.5 (IQR: 26.1–66.5) to 0.00 (IQR: 0.00–14.3) (p < 0.05). The mean halo eccentricity was 0.50 ± 0.08 degrees. Spectacle independence was reported by 22 (91.7%), 20 (83.3%), and 13 (54.2%) patients for distance, intermediate, and near tasks, respectively. Overall, 22 (91.7%) patients reported postoperative satisfaction.

**Conclusion:**

The AcrySof IQ Vivity IOL demonstrated favourable outcomes in patients with early or intermediate AMD, offering a meaningful range of spectacle-free vision with minimal photic phenomena.

## Introduction

Implantation of presbyopia-correcting multifocal (MF) IOLs in eyes with preexisting retinal pathology, such as age-related macular degeneration (AMD), remains a subject of ongoing debate. Concerns stem primarily from the optical compromises inherent in MF IOL designs, which achieve multifocality by splitting incoming light across multiple focal points using refractive, diffractive, or hybrid technologies. While effective in healthy eyes, these lenses may exacerbate visual disturbances such as glare, halos, and reduced contrast sensitivity, posing challenges in eyes with compromised macular function [1].

Rather than distributing light across distinct focal points, extended depth-of-focus (EDoF) IOLs are specifically engineered to extend the focal range by generating a continuous elongated focal zone, providing optimal uncorrected vision for distance, intermediate, and, to varying degrees, near tasks. This design is associated with improved contrast sensitivity and reduced photic phenomena when compared to traditional MF IOLs, making them potentially more favourable for individuals with macular impairment [2]. Nevertheless, the evidence on their use in eyes with macular pathology is limited, and a clear superiority of EDoF lenses’ quality of vision compared to monofocal IOLs has yet to be convincingly established [3, 4].

A recent advancement in the EDoF category is the AcrySof IQ Vivity IOL (Alcon, Fort Worth, TX), with its non-diffractive wavefront-shaping technology. This novel design aims to deliver an extended range of high-quality vision while preserving a photic profile closer to that of monofocal IOLs. The distinctive feature of this lens is its non-diffractive surface, engineered with X-WAVE technology. By means of two transition zones, it reshapes the incoming light wavefront to extend the depth of focus perceivable by the retina, without increasing patient reported dysphotopsias. Those two zones comprehend a slightly elevated plateau that delays part of the wavefront, stretching it to generate a continuous focal range and a subtle curvature change across a central 2.2 mm zone that shifts the wavefront forward, optimizing use of the available light energy. Early clinical studies have reported promising results in terms of visual acuity, contrast sensitivity, and patient satisfaction in the general cataract population [5, 6]. However, data regarding its outcomes in eyes with concurrent macular pathology, particularly early and intermediate stages of AMD, remain limited and inconclusive.

Given the growing popularity of EDoF lenses and the rising prevalence of AMD in the aging population, it is crucial to evaluate the performance of emerging IOL technologies in the context of existing or potential macular pathology.

The present study aims to assess the visual outcomes, quality of vision, and patient-reported satisfaction following the bilateral implantation of the AcrySof IQ Vivity IOL in individuals diagnosed with early or intermediate AMD.

## Methods

This prospective, single-centre study was conducted at the Sant'Anna University Hospital, University of Ferrara, Italy, between March 2024 and March 2025. This study adhered to the tenets of the Declaration of Helsinki and was approved by the institutional Ethics Committee. All patients signed an informed consent before enrolment, and all data were collected anonymously.

Patients aged 55 years or older with bilateral age-related cataract and bilateral early or intermediate stage AMD (eAMD and iAMD, respectively) were screened for enrolment. eAMD and iAMD were defined as follows [7]:

- eAMD: medium sized drusen (≥ 63 and < 125 μm), but without pigmentary abnormalities thought to be related to AMD,
- iAMD: large drusen (≥ 125 μm) or pigmentary abnormalities associated with at least medium drusen

Exclusion criteria were the presence of (1) corneal astigmatism > 1.00 D, (2) pupil diameter > 4.5 mm, (3) angle kappa > 0.5 mm, (4) amblyopia, (5) other visually impairing media opacities (e.g. corneal leukoma), (6) glaucoma, (7) macular pathologies other than eAMD or iAMD including neovascular and atrophic AMD, (8) and the development of complications during surgery influencing the visual outcome or requiring the implant of a different type of IOL.

Eligible patients underwent a preoperative evaluation under photopic condition consisting of (1) monocular and binocular distance-corrected and unaided visual acuity at far, intermediate, and near distance, (2) clinical and tomographical AMD staging, (3) dysphotopsia perception evaluated through the McAllinden’s Quality of Vision (QoV) Questionnaire, (4) visual subjective performance evaluated through the Catquest-9SF Questionnaire, (5) and ocular biometry using the Zeiss IOL Master 700 (Carl Zeiss Meditec, Jena, Germany).

Visual acuity measurements were assessed under photopic condition in logarithm of the minimum angle of resolution (logMAR) using early treatment diabetic retinopathy study (ETDRS) charts specifically designed for each testing distance: far (4 m), intermediate (66 cm), and near (40 cm).

IOL power calculation was performed using the Barrett Universal formula. Among the IOL powers predicted to result in a myopic refraction, the one giving the least negative target was selected.

In all patients, both eyes were operated using the same technique. A time interval of 4 weeks elapsed between the first and second procedure.

All surgeries were performed using the Centurion Vision System phacoemulsifier (Alcon Laboratories, Inc., Fort Worth, TX, USA) under topical anaesthesia, with the surgeon seated at the head of the patient. A direct chop phacoemulsification technique was employed, utilizing a superotemporal clear corneal incision of 2.2 mm and two side port incisions at 2–3 o’clock hours on either side of the main wound. The lens was then implanted into the posterior chamber within the capsular bag. Postoperatively, all patients underwent a comprehensive ophthalmological examination at 1 week, 1 month, and 3 months after surgery. 3-month data represent the primary outcome timepoint.

The primary endpoint of this study was monocular corrected distance visual acuity (mCDVA). Secondary outcomes included (1) monocular uncorrected distance visual acuity (mUDVA), (2) binocular corrected and unaided distance visual acuity (bCDVA and bUDVA, respectively), (3) monocular and binocular distance-corrected and unaided intermediate (mDCIVA, bDCIVA, mUIVA, bUIVA, respectively) and (4) near visual acuity (mDCNVA, bDCNVA, mUNVA, bUNVA, respectively); (5) monocular defocus curve was assessed in logMAR using ETDRS Charts at 4 m with 0.25D steps between ± 0.50D and 0.50D steps in the rest of the range, (6) postoperative QoV Questionnaire to assess the perception of bothering dysphotopsias, (7) postoperative Catquest-9SF Questionnaire Score to evaluate the visual performance and satisfaction of the patients, (8) spectacle-independence measured through the IOLSAT Questionnaire, (9) and objective haloes quantification through the Aston Halometer (Aston University, Birmingham, UK) [8].

Furthermore, all patients enrolled in the study were asked, both prior to and following the surgical procedures, how much they were satisfied with their vision (very unsatisfied, unsatisfied, satisfied, very satisfied).

### Statistical Analysis

Microsoft Excel (Microsoft, Redmond, WA, USA) and GraphPad Prism 8 (GraphPad Software, San Diego, CA, USA) were used for data collection and statistical analysis, respectively.

To determine the required sample size for the study, an a priori power analysis was performed based on the results from the study of Rementería-Capelo et al. [9] Based on their data, assuming a standard deviation for BCVA of 0.28 logMAR, a sample of 28 patients was required to detect a mean change of 0.18 logMAR in BCVA, with a power of 0.95 and P value of 0.05. However, to ensure adequate reliability and account for a plausible 10% dropout rate, we aimed for a sample size of 30 patients.

For monocular measurements and analysis, one eye per patient was selected using simple randomization with a computer-generated random number sequence to avoid pseudoreplication bias due to the non-independence of observations from both eyes of the same subject. The choice between the right and left eye was made randomly to prevent potential systematic bias that could arise from always selecting the same eye.

Patient-reported outcomes assessed using the QoV and Catquest-9SF questionnaires were converted from raw ordinal scores to interval-level measures using an approximate Rasch-based transformation, and subsequently rescaled to a 0–100 scale with higher scores indicating greater visual disturbance or disability.

Normality was assessed using the Shapiro–Wilk test. Halometry measurements, which were found to be normally distributed, were summarized using the mean and standard deviation. Paired t-test was conducted to compare preoperative and postoperative results.

The other variables were described by the median and interquartile range, and the non-parametric Wilcoxon signed-rank test was used for comparative analysis.

The McNemar test was used to assess any significant difference between the preoperative and postoperative prevalence of each dysphotopsia as reported in the QoV Questionnaire, and in the percentages of patients satisfied with their vision.

## Results

A total of 30 patients were enrolled. During the study, three patients developed health issues unrelated to the eye and withdrew from the trial; one patient was lost to follow-up; two patients were excluded due to posterior capsular rupture during surgery and three-piece IOL implantation.

A total of 24 patients (48 eyes) completed the required follow-up visits. The mean age was 74.5 ± 5.5 years, and 12 (50%) were male.

**Fig. 1:**
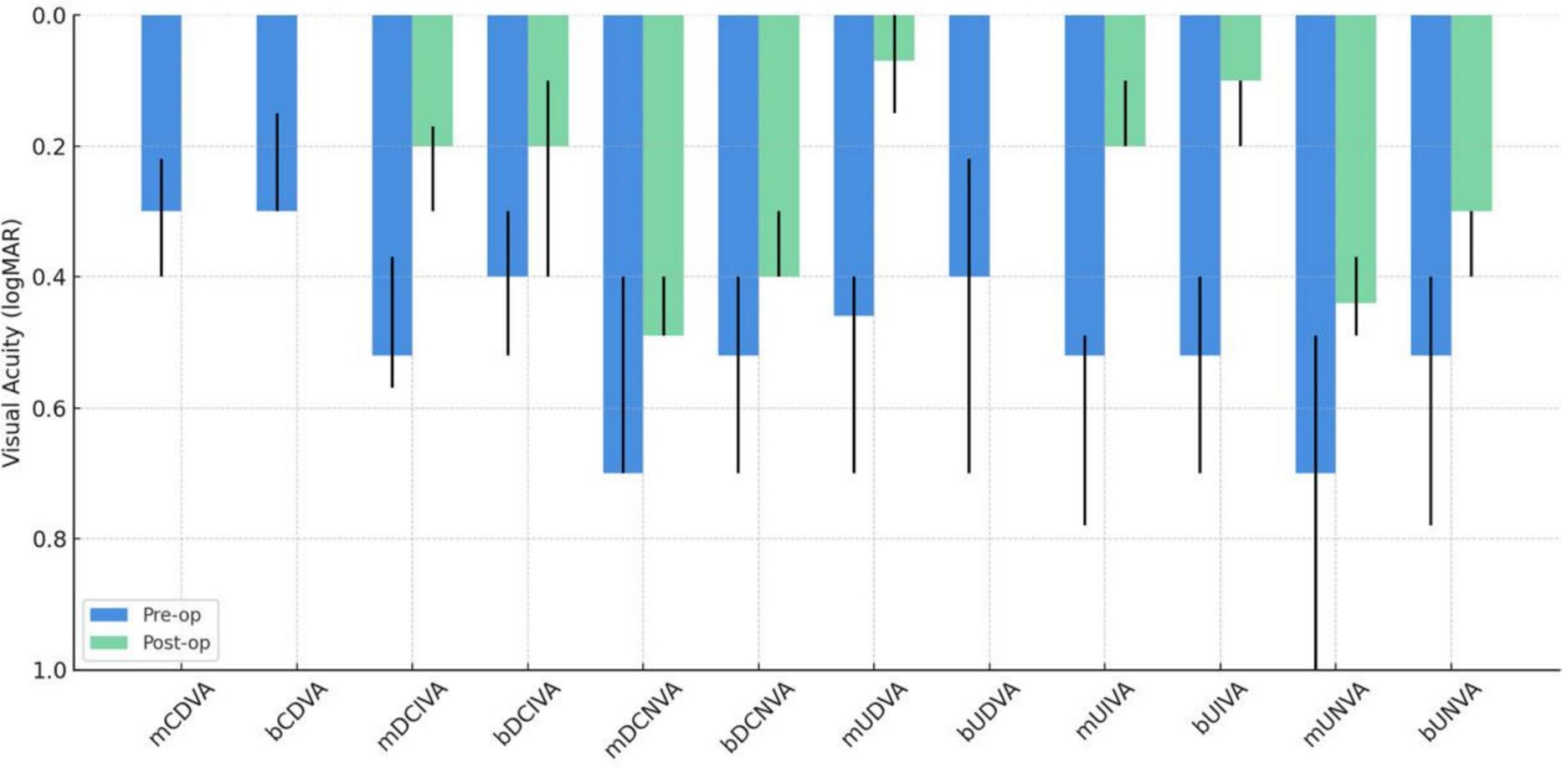
Preoperative and 3 months postoperative visual acuities at far, intermediate, and near distance (median ± IQR)

Median preoperative and 3-month postoperative corrected and unaided monocular and binocular visual acuity at far intermediate, and near distance are reported in Table 1 and Fig. 1. There was a statistically significant postoperative improvement in visual acuity at each distance tested (p < 0.05). The mean post-operative residual spherical equivalent was 0.34 ± 0.26 D.

**Table 1:** Preoperative and 3 months postoperative visual outcomes

	Pre-op median value (interquartile range)	3-month Post-op median value (interquartile range)	Difference (% of change)	p-value
mCDVA	0.30 (0.22–0.40)	0.00 (0.00–0.00)	0.30 (100%)	< 0.001*
bCDVA	0.30 (0.15–0.30)	0.00 (0.00–0.00)	0.30 (100%)	< 0.001*
mDCIVA	0.52 (0.37–0.57)	0.20 (0.17 – 0.30)	0.32 (61.4%)	< 0.001*
bDCIVA	0.40 (0.30–0.52)	0.20 (0.10 – 0.40)	0.20 (49.8%)	< 0.001*
mDCNVA	0.70 (0.40–0.70)	0.49 (0.40 – 0.49)	0.21 (29.2%)	< 0.05*
bDCNVA	0.52 (0.40–0.70)	0.40 (0.30 – 0.40)	0.12 (23.7%)	< 0.001*
mUDVA	0.46 (0.40–0.70)	0.07 (0.00 – 0.15)	0.39 (84.0%)	< 0.001*
bUDVA	0.40 (0.22–0.70)	0.00 (0.00 – 0.00)	0.40 (100%)	< 0.001*
mUIVA	0.52 (0.49–0.78)	0.20 (0.10 – 0.20)	0.32 (61.6%)	< 0.001*
bUIVA	0.52 (0.40–0.70)	0.10 (0.10 – 0.20)	0.42 (81.4%)	< 0.001*
mUNVA	0.70 (0.49–1.00)	0.44 (0.37 – 0.49)	0.26 (36.5%)	< 0.001*
bUNVA	0.52 (0.40–0.78)	0.30 (0.30 – 0.40)	0.22 (42.4%)	< 0.001*

mCDVA: monocular corrected distance visual acuity; bCDVA: binocular corrected distance visual acuity; mDCIVA: monocular distance corrected intermediate visual acuity; bDCIVA: binocular distance corrected intermediate visual acuity; mDCNVA: monocular distance corrected near visual acuity; bDCNVA: binocular distance corrected near visual acuity; mUDVA: monocular uncorrected distance visual acuity; bUDVA: binocular uncorrected distance visual acuity; mUIVA: monocular uncorrected intermediate visual acuity; bUIVA: binocular uncorrected intermediate visual acuity; mUNVA: monocular uncorrected near visual acuity; bUNVA: binocular uncorrected near visual acuity

^*^P < 0.05

**Fig. 2:**
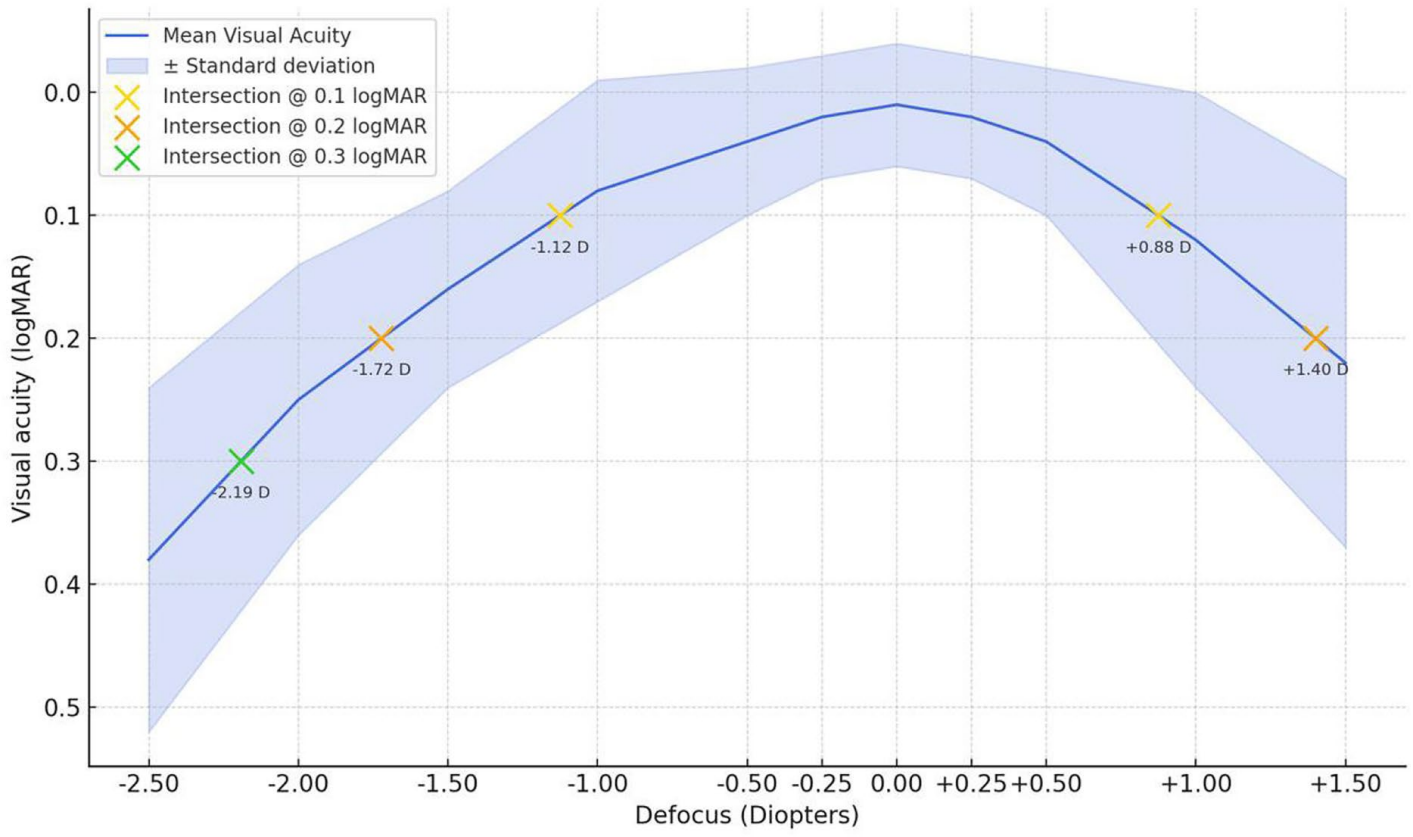
Schematic representation of the monocular defocus curve from -2.50 D to + 1.50 D 3 months postoperative. The yellow, orange, and green crosses indicate the defocus diopter values at which visual acuities of 0.1, 0.2, and 0.3 logMAR are respectively intersected

Figure 2 shows the mean monocular defocus curve from -2.50D to + 1.50D 3 months after surgery. The yellow, orange, and green crosses indicate the defocus diopter values at which visual acuities of 0.1, 0.2, and 0.3 logMAR are respectively intersected. As shown in the graph, the cohort obtained a mean visual acuity ≤ 0.1 logMAR in the range spanning from -1.12 to + 0.88 diopters of defocus, a mean visual acuity of ≤ 0.2 logMAR in the range from -1.72 to + 1.40 diopters, and a mean visual acuity of ≤ 0.3 logMAR in a range from -2.19 to + 1.50 diopters.

**Fig. 3:**
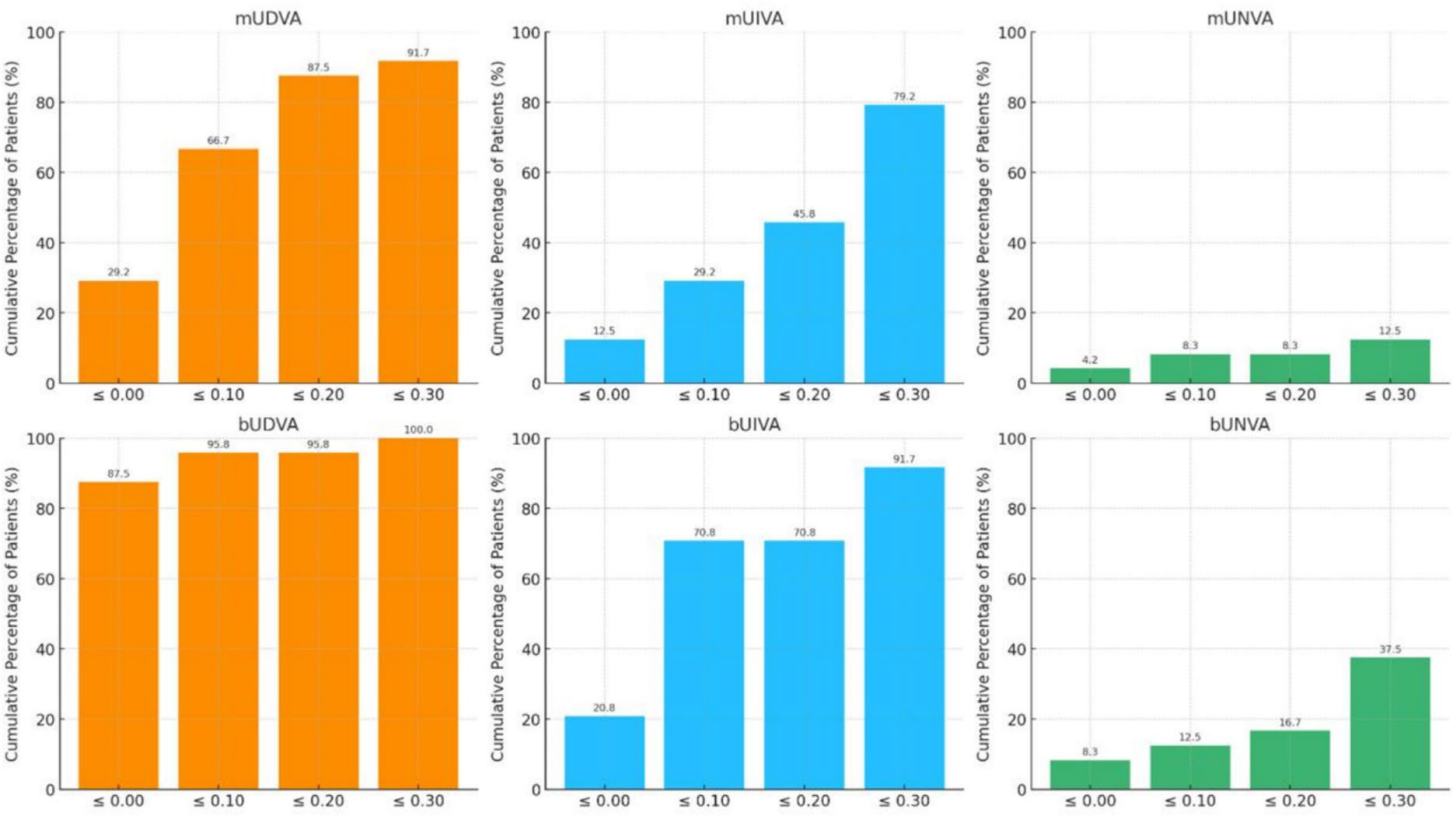
Cumulative percentage of patients achieving different threshold values of uncorrected distance, intermediate, and near visual acuity (logMAR) 3 months postoperative

Figure 3 illustrates the cumulative percentage of patients achieving different levels of monocular and binocular unaided visual acuity at each measured distance (distance, intermediate, and near) 3 months after surgery. A satisfactory functional binocular unaided visual acuity of ≤ 0.2 logMAR was obtained by 23 (95.8%) and 17 (70.8%) subjects at far and intermediate distances, respectively, whereas 4 (16.7%) patients achieved spectacle-free vision of ≤ 0.2 logMAR at near distances.

### Aston Halometry

The mean halo eccentricity from the central stimulus, measured along eight meridians 45° apart, was 0.50 ± 0.08 degrees, with a mean halo area of 0.72 ± 0.21 degs^2^.

### Questionnaires Results

Table 2 shows the prevalence of each dysphotopsia reported pre- and postoperatively using the QoV questionnaire 3 months after surgery. A significant postoperative reduction was observed across all visual disturbances (p < 0.001), with the most notable decreases occurring in glare (20 vs 4, 83.3% vs 16.7%), starburst (19 vs 6, 79.2% vs 25.0%), and blurred vision (17 vs 1, 70.8% vs 4.2%).

**Table 2:** Preoperative and 3 months postoperative prevalence of different types of dysphotopsia as reported in the QoV Questionnaire

Dysphotopsia Type	% Pre-Op	% Post-Op	p-value
Glare	83.3	16.7	< 0.001
Haloes	62.5	4.2	< 0.001
Starburst	79.2	25.0	< 0.001
Hazy Vision	70.8	8.3	< 0.001
Blurred Vision	70.8	4.2	< 0.001
Distortion	33.3	8.3	< 0.001
Multiple images	20.8	0.0	< 0.001
Fluctuation in vision	20.8	0.00	< 0.001
Focusing difficulties	79.2	8.3	< 0.001
Distance/depth perception difficulties	41.7	4.2	< 0.001

**Fig. 4:**
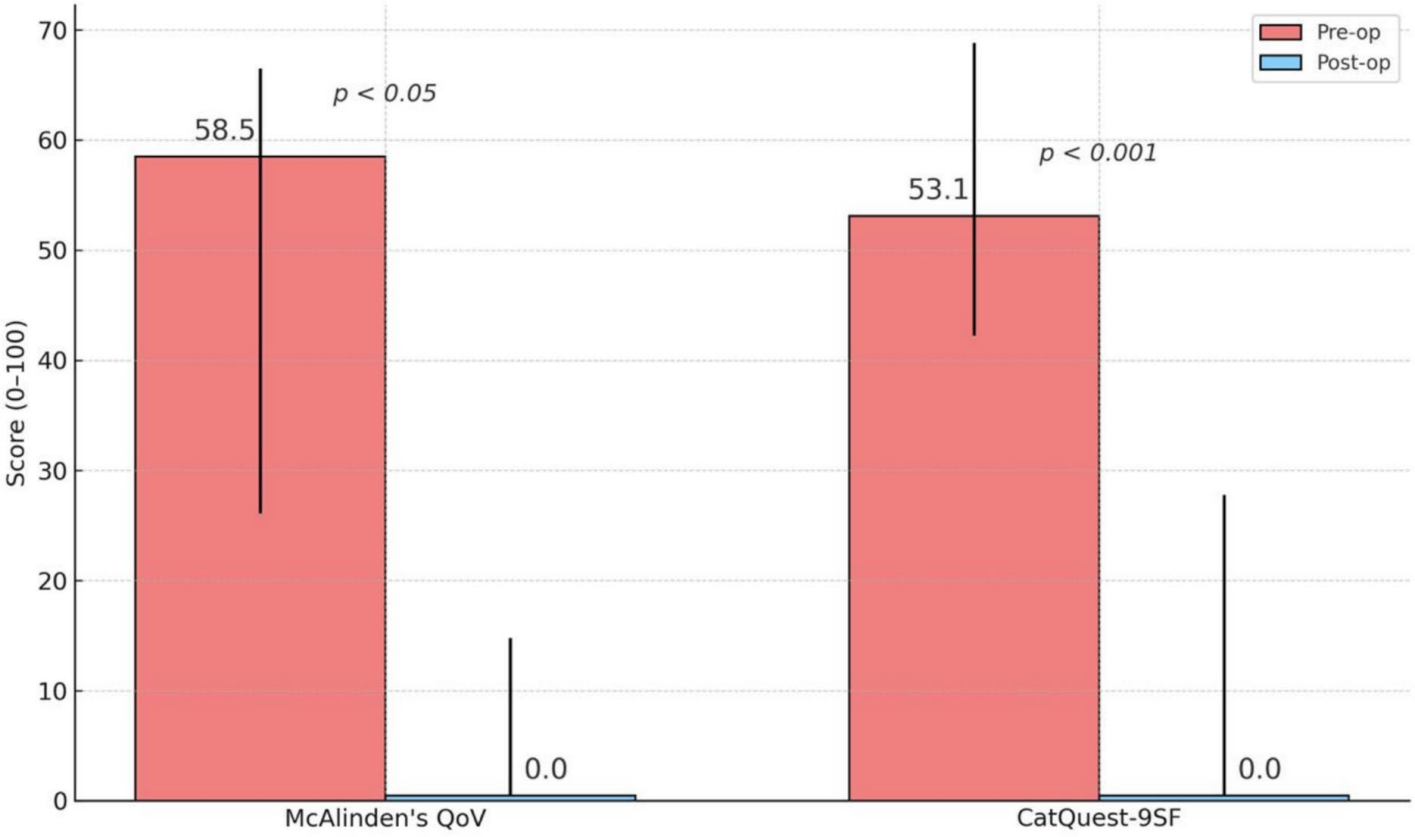
Preoperative and 3 months postoperative score of the McAlinden’s QoV and CatQuest-9SF Questionnaires (0–100 scale)

At 3 months after surgery, a significant improvement was observed in the scores of both the QoV and CatQuest-9SF questionnaires, resumed in the Fig. 4.

The median QoV score significantly decreased from 58.5 (IQR: 26.1–66.5) to 0.00 (IQR: 0.00–14.3) after IOL implantation (p < 0.05). Similarly, the degree of visual function satisfaction expressed by the CatQuest-9SF score showed a significant improvement from a median of 53.1 (IQR: 42.2–68.8) to 0.0 (IQR: 0.0–27.3) (p < 0.001).

Based on the responses to the IOLSAT Questionnaire, the percentages of subjects never or rarely needing glasses were 22 out of 24 (91.7%), 20 out of 24 (83.3%), and 13 out of 24 (54,2%) for distant, intermediate, and near everyday activities, respectively.

**Fig. 5:**
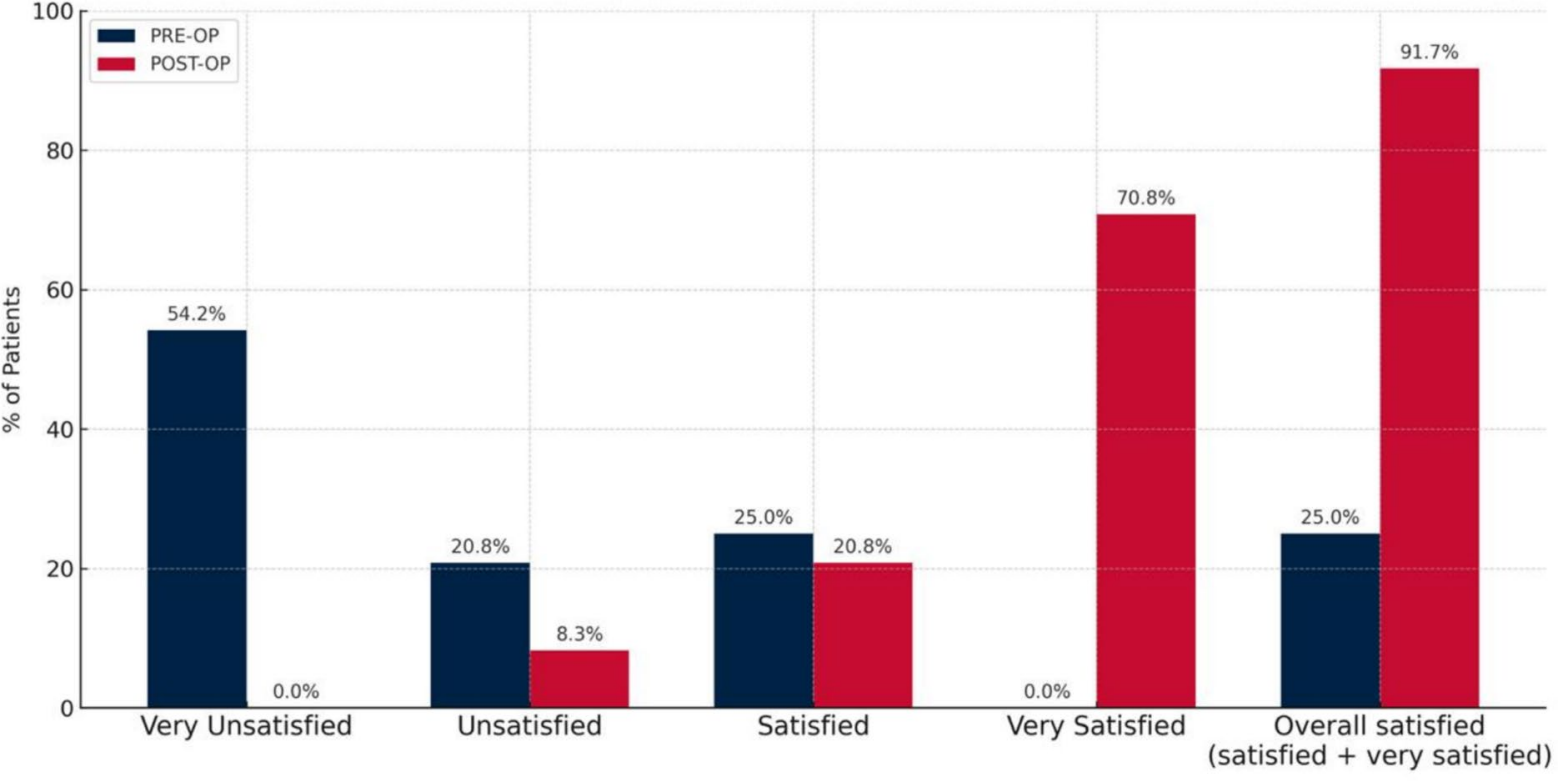
Preoperative and 3 months postoperative cumulative percentages of patients satisfied with their vision

Figure 5 shows the cumulative percentages of people satisfied with their vision pre- and 3 months postoperatively, which experienced a significant increase from 6 out of 24 (25.0%) preoperatively to 22 out of 24 (91.7%) postoperatively (χ^2^ = 16.0, p < 0.001).

## Discussion

This prospective single-center study represents the first report in the literature of the functional outcomes of bilateral AcrySof IQ Vivity IOL implantation in patients with eAMD or iAMD.

Our results demonstrated that the Vivity IOL may provide significant improvements in monocular visual acuity at distance, intermediate, and near ranges, with a low incidence of dysphotopsias and high patient satisfaction levels (all p < 0.05).

Similarly, at 3 months postoperatively, 95.8% of patients achieved a bUDVA of ≤ 0.2 logMAR, while 70.8% reached the same level of visual acuity at intermediate distances. Although near vision performance was comparatively limited, with only 16.7% of subjects achieving a logMAR score of 0.2 or better, the overall range of functional vision was clinically meaningful. Moreover, the IOLSAT Questionnaire scores supported our findings on spectacle independence, with 91.7% and 83.3% of participants reporting that they never or rarely needed glasses for distance and intermediate tasks, respectively. Our visual outcomes are consistent with prior studies on the Vivity IOL in both AMD and non-AMD populations, suggesting that this non-diffractive EDoF design can offer a balanced compromise between range of vision and optical quality, particularly in eyes with mild macular dysfunction [10–12].

The monocular defocus curve at 3 months postoperatively demonstrated a continuous range of functional vision, with a mean visual acuity of ≤ 0.2 logMAR from + 1.40 D to –1.72 D, confirming the robust intermediate performance and degree of functional near vision provided by this lens and aligning with previously described defocus curves obtained by the Vivity IOL in healthy subjects [13]. Importantly, while the near performance does not match that of multifocal IOLs, the smoother defocus curve may offer distinct advantages, especially in patients with macular impairment. These results align with patients’ growing expectations for reduced dependence on glasses, even in AMD cohorts, and support strong performance in intermediate-distance daily tasks such as computer use. Regarding the 54.2% of patients reporting spectacle independence for near-distance activities in the IOLSAT Questionnaire, the observed outcome is consistent with the optical design of the lens and was therefore expected, highlighting the importance of managing expectations in patients with AMD, especially concerning near vision performance [14].

Importantly, Vivity IOL implantation was associated with a significant reduction in each photic phenomenon described in the QoV questionnaire, whose median score showed a significant postoperative improvement from 58.5 to 0.0. Objective measurements using the Aston Halometer corroborated these subjective findings, showing tolerable halo formation with a mean eccentricity of 0.50 ± 0.08 degrees, comparable to those described in the literature for monofocal IOLs [15].

A favorable photic profile, such as that observed with the Vivity IOL in our study, is particularly relevant in patients with retinal compromise, where minimizing visual disturbances is crucial for maintaining quality of life and achieving high patient satisfaction, as observed in this study and previous reports [11]. This study provides valuable insights into a patient population that is often excluded from clinical trials involving presbyopia-correcting IOLs. With the increasing prevalence of AMD and cataract and with rising demand for spectacle independence, careful expansion of EDoF indications to include early and intermediate AMD may improve postoperative satisfaction and visual function in selected patients.

This study is limited by its relatively small sample size, single-center design, lack of a control group and the absence of stratification of eAMD and iAMD population based on OCT scans. In addition, the assessments were done under photopic conditions only and did not evaluate contrast sensitivity. Future studies incorporating formal contrast sensitivity and mesopic testing are warranted to substantiate the potential benefits observed in our study. However, given the Vivity IOL’s non-diffractive design able to provide a more uniform wavefront with minimal scatter, patients may be able to retain contrast sensitivity more than diffractive multifocal IOLs, as shown in previous reports comparing contrast sensitivity after Vivity IOL and monofocal lenses implantation [13].

Furthermore, the limited data available from the literature regarding the Vivity IOL impact on contrast sentivity in AMD patient revealed a reduction pattern primarily due to AMD progression rather than IOL implantation [6], supporting the idea that this new design of EDOF lens may not influence contrast sensitivity negatively even in patients with impaired macular function. Additionally, by providing a range of spectacle-free vision while preserving contrast sensitivity, the non-diffractive EDOF IOL may be a promising option for patients with AMD. Furthermore, the short follow-up period precludes conclusions regarding long-term performance, especially in the case of AMD progression. In this regard, comparison with data from a recent Australian pilot study suggests that while EDoF lenses may offer benefits in early stages of disease, these benefits may decline as macular impairment progresses. In addition, the short follow-up time may limit the visual recovery and tolerability following the implant of this particular type of lenses, as neuroadaptation may take longer than our 3-months follow-up to fully take place. [16]

Future studies with larger populations, longer follow-up, and randomized comparative arms taking into consideration also contrast sensitivity, mesopic conditions, and OCT-based AMD staging and stratification will help validate and extend our findings further. Finally, secondary outcomes were analysed without adjustment for multiplicity; reported p-values are exploratory.

In conclusion, the AcrySof IQ Vivity IOL appears to be an effective option for patients with early or intermediate AMD. With appropriate preoperative patient selection and counseling, this lens can offer a meaningful improvement in intermediate and distance vision with minimal dysphotopsias in a population historically considered suboptimal for presbyopia-correcting implants.

## Data Availability

Data will be made available upon reasonable request.
